# Malignancy-Induced Functional Tricuspid Obstruction Complicated by Cardiopulmonary Collapse Treated Using Combination VA-ECMO and AngioVac

**DOI:** 10.1016/j.jaccas.2025.103692

**Published:** 2025-04-02

**Authors:** Hafez Golzarian, Anna C. Kleman, Mallory Knous, Janelle Fields, Dylan Pitts, Andria L. Utendorf, Amanda Laird, Aakash Kumar, William C. Cole, Robert A. Baiocchi, Sandeep M. Patel

**Affiliations:** a Internal Medicine Residency Program, BonSecours Mercy Health–St. Rita’s Medical Center, Lima, Ohio, USA; b Department of Cardiovascular Disease, HCA Houston–Kingwood/University of Houston College of Medicine, Houston, Texas, USA; c Structural Heart and Intervention Center, BonSecours Mercy Health–St. Rita’s Medical Center, Lima, Ohio, USA; d Department of Critical Care, BonSecours Mercy Health–St. Rita’s Medical Center, Lima, Ohio, USA; e Division of Hematology and Oncology, The James–Ohio State University’s Wexner Medical Center, Columbus, Ohio, USA

**Keywords:** AngioVac, cardiac arrest, extracorporeal membrane oxygenation, lymphoma, malignancy, shock, thrombectomy, thrombus

## Abstract

**Background:**

Right-sided intracardiac masses, often arising from metastatic malignant diseases infiltrating the central venous system, may cause profound hemodynamic instability and life-threatening obstructive shock.

**Case Summary:**

The authors present a case demonstrating the extent of both diagnostic and therapeutic utility of percutaneous large-bore vacuum-assisted aspiration thrombectomy in a patient presenting with intracardiac tumor-induced tricuspid valve obstruction and shock with subsequent cardiac arrest. Our patient was successfully resuscitated with venoarterial (VA) extracorporeal membrane oxygenation (ECMO) with a bridge to aspiration of the large intracardiac obstructing tumor by using a large-bore vacuum-assisted aspiration thrombectomy system.

**Discussion:**

This case demonstrates the successful use of large-bore aspiration thrombectomy in conjunction with VA ECMO to manage a patient presenting with a large intracardiac mass and obstructive shock. Large-bore vacuum-assisted aspiration thrombectomy facilitated both hemodynamic stabilization and rapid tissue diagnosis, which was crucial for guiding further management.

**Take-Home Message:**

Although aggressive intervention in such cases may initially seem unorthodox, particularly in a patient with suspected metastatic disease, this case highlights its potential benefits when combined with a multidisciplinary approach and strong consideration of patient and family goals of care.

## Graphical Abstract

**Figure:**
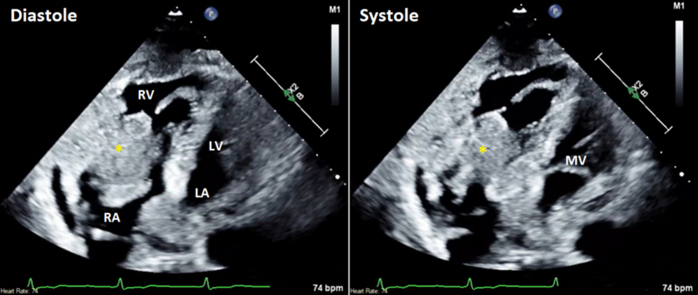

Diagnosis and staging of cancer typically involve various imaging techniques and tissue biopsies to identify the presence, location, and characteristics of cancerous cells. In various cases of metastatic disease, tumor cells may infiltrate the central venous system and result in formation of right-sided intracardiac masses that could propagate profound obstructive hemodynamic instability.^1^^,^^2^ We report the case of a patient who presented with obstructive shock secondary to metastasis of an unknown aggressive malignant disease that resulted in nearly total obstruction of her tricuspid valve. We were able to resuscitate her with venoarterial (VA) extracorporeal membrane oxygenation (ECMO), followed by the percutaneous debulking of her intracardiac mass with the AngioVac aspiration system (AngioDynamics). This allowed us to obtain a definitive diagnosis and staging of metastatic aggressive diffuse large B-cell lymphoma (DLBCL) and facilitate her care with an oncologist. Cardiac masses, whether primary or metastatic, are not as uncommon as once thought, and the safety and feasibility of large-bore vacuum-assisted aspiration thrombectomy systems elucidate their potential diagnostic utility analogous to that of conventional biopsies, even in patients with aggressive oncologic burden.

**Take-Home Messages**

• Large-bore vacuum-assisted aspiration thrombectomy in combination with VA ECMO may provide a feasible and synergistic means of resuscitating critically ill patients with malignancy-induced cardiopulmonary collapse.
• Clinicians should individualize risk-benefit assessments when considering large-bore vacuum-assisted aspiration thrombectomy systems in the setting of hemodynamic instability or late-stage malignant disease.
• Multidisciplinary collaboration in managing complex cardiovascular complications of malignancy is paramount in optimizing clinical outcomes.

## History of Presentation

**Figure 1:**
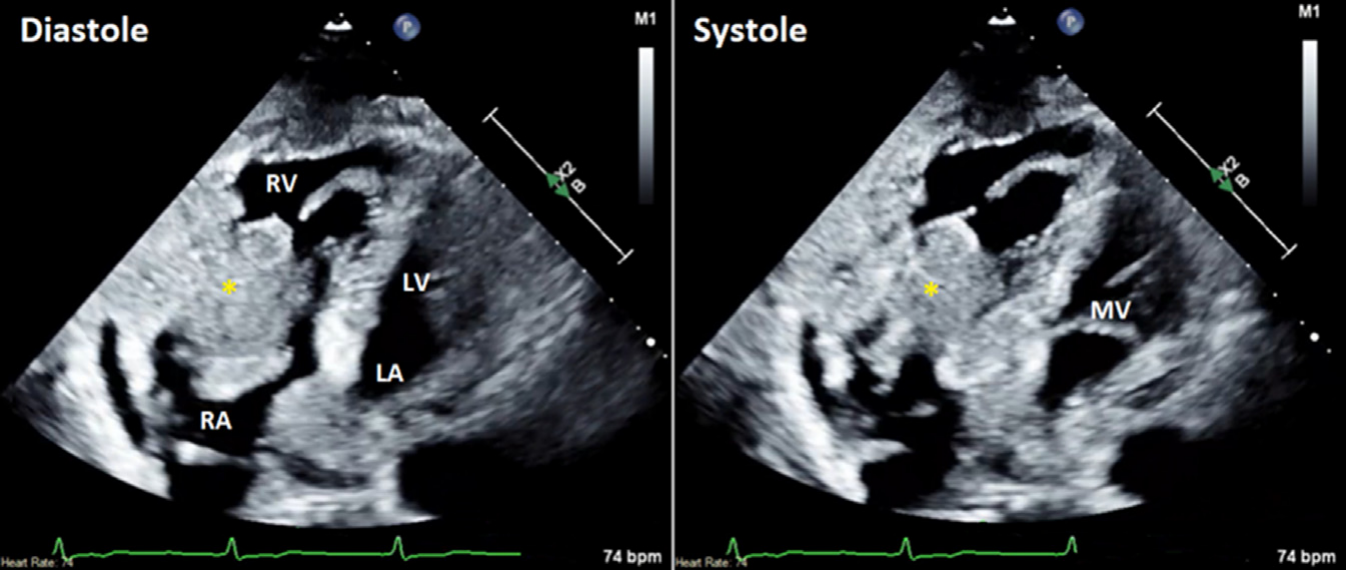
Transthoracic Echocardiogram (Subcostal 4-Chamber View) The yellow asterisk denotes a large tumorlike mass with subtotal tricuspid obstruction. LA = left atrium; LV = left ventricle; MV = mitral valve; RA = right atrium; RV = right ventricle.

A 66-year-old woman without a previous history presented with new onset progressive fatigue, malaise, and unintentional weight loss. She was hypotensive, with a mean arterial pressure of 41 mm Hg. On physical examination, she had altered mental status and grade IV jugular venous distention. Relevant laboratory findings included a white blood cell count of 16.2 × 10^3^ cells/μL, a lactate level of 8.4 mmol/L, a troponin level of 911 ng/L, and an N-terminal pro–B-type natriuretic peptide level of 13,284 pg/mL. Bedside transthoracic echocardiography demonstrated a large right atrial mass that subtotally obstructed the tricuspid annulus with mean gradient of 9 mm Hg (Figure 1, Video 1A).

**Figure 2:**
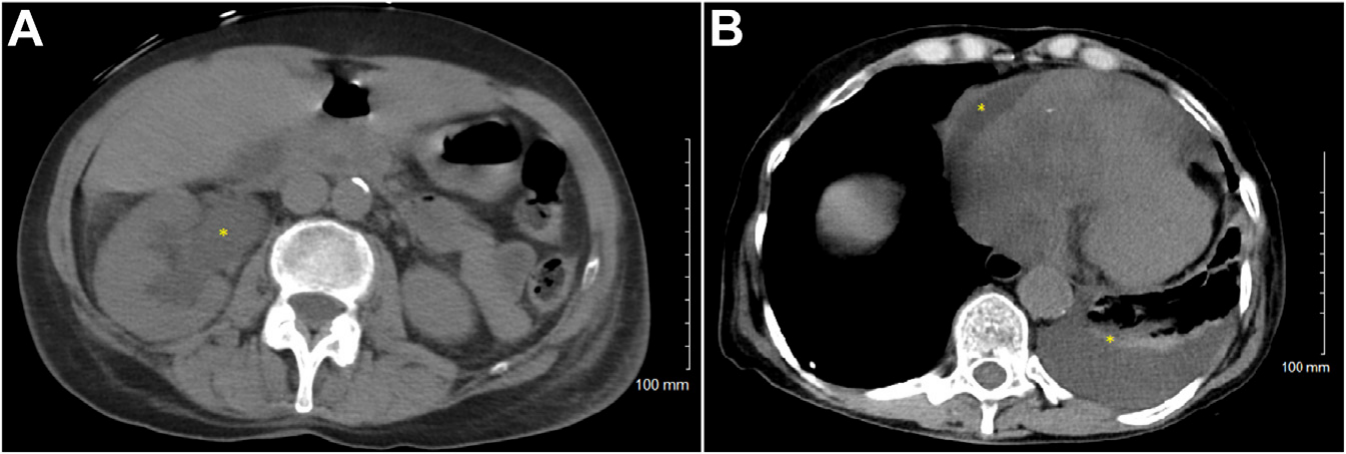
Computed Tomography of the Abdomen (A)The right-sided hydronephrosis and mass at the renal pelvis suggestive of malignancy. (B) Neoplastic changes in the mediastinum, pericardial thickening, and a left lower lobe pleural effusion that appears exudative and is suggestive of a metastatic process.

The structural heart team was promptly paged, and emergency transesophageal echocardiography confirmed nearly complete diastolic obstruction of the tricuspid valve with a mass measuring 5.8 cm × 4.1 cm (Video 2A). Incidental computed tomographic imaging revealed additional concerning findings, including right-sided hydronephrosis from an irregularly shaped mass, ascites, and neoplastic changes involving the mediastinum, pericardium, soft tissues of the neck, and vena cava, thus raising suspicion for late-stage malignant disease (Figures 2A and 2B).

Obstructive shock was presumed, prompting aggressive intravenous fluid resuscitation and norepinephrine administration. After discussion with the family, a multidisciplinary decision was made to attempt percutaneous debulking and aspiration of the intracardiac mass on an emergency basis by using the percutaneous large-bore vacuum-assisted aspiration thrombectomy system to allow for definitive oncologic diagnosis. However, as the initial sheath was inserted, the patient’s condition continued to deteriorate, leading to cardiac arrest (pulseless electrical activity).

## Intervention

**Figure 3:**
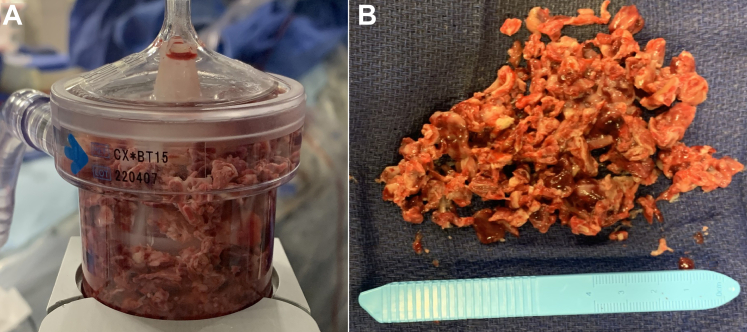
Intracardiac Debulking Tissue Sample Obtained (A) The collected tissue sample post filtration through the AngioVac (AngioDynamics) circuit. (B) The cumulative tumor burden debulked by the conclusion of the procedure.

As cardiopulmonary resuscitation was initiated, we promptly instituted VA ECMO to stabilize the patient. We converted the left groin access to a 19-F arterial return cannula with antegrade 6-F femoral access for external femoral bypass and obtained right femoral vein access with a 25-F multistage venous drainage cannula. Thereafter, we connected a standard VA ECMO circuit to provide ongoing cardiac support. We then obtained right internal jugular and left femoral venous access. The jugular vein was dilated to accommodate a 26-F Gore DrySeal sheath (W.L. Gore & Associates), and the left femoral vein was dilated to a 19-F venous return cannula. We then used the large-bore vacuum-assisted aspiration thrombectomy system to meticulously aspirate the large intracardiac mass (Figures 3A and 3B), with complete removal of the mass confirmed under echocardiographic visualization (Video 2B). A sample of the aspirated mass was sent for pathologic analysis.

Over the subsequent days, the patient required pericardiocentesis, aggressive ventilatory support, and large-volume diuresis. The patient demonstrated an excellent response to ECMO weaning trials and was liberated from the ECMO circuit on the sixth day. Histopathologic examination of the mass revealed DLBCL. The patient was ultimately extubated, stabilized, and then transferred for intensive rituximab, cyclophosphamide, doxorubicin (hydroxydaunomycin), vincristine (Oncovin), and prednisone (R-CHOP) chemotherapy, to which she had an excellent response.

## Follow-Up

Our patient is now 6 months post cardiac arrest and doing well without any further cardiopulmonary or neurologic limitations to date. She has thus far tolerated all immunochemotherapy sessions well and is anticipating complete remission in the upcoming months. Her follow-up echocardiograms demonstrated complete normalization of the pericardium and cardiac structures (Video 1B).

## Discussion

The AngioVac system, approved by the U.S. Food and Drug Administration in 2014 for the removal of unwanted intravascular material, consists of a venous drainage cannula and a reinfusion venous return cannula connected to an extracorporeal circuit, filter, and bypass pump.^3^ This setup creates a vacuumlike effect that facilitates the aspiration of blood and vascular content into the cannula, which then filters the blood and returns it through the reinfusion venous cannula. In recent years, the utility of these large-bore vacuum-assisted aspiration thrombectomy systems has expanded to include use for debulking vegetations and various masses.^4^^,^^5^ These systems have demonstrated versatility in managing thrombus and tumor burdens within various cardiac chambers and vasculature, including left-sided masses such as those found on the mitral valve, in the left ventricle, and in the aortic arch.

This case report demonstrates both the diagnostic and the therapeutic potential of the large-bore vacuum-assisted aspiration thrombectomy systems in the management of large intracardiac masses and obstructive shock. Given the patient’s unstable condition, which required prompt intervention, surgical intervention was not feasible. Percutaneous large-bore vacuum-assisted aspiration thrombectomy using the AngioVac system represented a suitable option because its large-diameter funnel enabled rapid aspiration, a critical factor given the urgency of the hemodynamic instability. Additionally, the system’s ability to return the aspirated blood was also crucial in the context of the patient’s hemodynamic compromise.

Several adjunctive maneuvers involving snares and balloons can complement large-bore vacuum-assisted aspiration thrombectomy systems. The snare technique involves a secondary access with a catheter snare to capture the mass, stabilize it, and allow for aspiration from the primary access site. Additionally, the snare catheter can be advanced via the Tuohy-Borst valve attachment of the main aspiration catheter through the funnel and allow for more central engagement of the funnel to the mass by capturing the mass with the snare. Balloons can be advanced next to the mass and inflated to hold the mass against the cardiac wall to allow for advancement of the aspiration catheter toward the mass. Finally, a long polypropylene (Prolene, Ethicon) suture can be placed on the inner curvature of the funnel, externalized, and pulled on the funnel from the outside to enable further direction of the aspiration catheter to the mass. However, in the case of our patient, these complementary techniques were not needed.

Although health care providers may hesitate to use large-bore vacuum-assisted aspiration thrombectomy systems in the setting of hemodynamic instability or metastatic disease because of the perceived lack of mortality benefit, this case report provides valuable insights for future risk-benefit assessments. Central to the management of this patient was prompt recognition of the impending obstructive cardiac arrest. The intracardiac mass was clearly obstructing filling of the right ventricle and resulting in a secondary “vena cava syndrome” with elevated venous pressures in both the superior and inferior vena cava. Although these vacuum-assisted aspiration thrombectomy systems require large-bore venous access, we were able to convert our accesses to VA ECMO cannulas and still proceed in providing therapy from the jugular site. This case highlights the importance for proceduralists to be aware of these types of intracardiac mass-related emergencies and the need to prepare for cardiopulmonary collapse. By prioritizing various access sites and using them effectively, proceduralists can address the underlying cause of the obstruction while simultaneously managing any anticipated hemodynamic instability.

Additionally, the large-bore vacuum-assisted aspiration thrombectomy system in this case enabled a clear diagnostic pathway. Although large-bore vacuum-assisted aspiration thrombectomy systems are typically used to remove unwanted vascular material, the large volume of aspirated tissue in this instance allowed for comprehensive histopathologic evaluation, facilitating a definitive diagnosis and informing the patient’s subsequent management. Without the use of the large-bore vacuum-assisted aspiration thrombectomy system, obtaining a definitive diagnosis could have required additional testing and procedures, potentially delaying treatment.

We acknowledge that aggressive interventions for patients with suspected metastatic disease may seem heretical and unorthodox. Clinicians should exercise prudence and avoid allowing less favorable diagnostic findings, such as the right renal mass with inferior vena cava thrombi observed in this case, to unduly influence their clinical decision making. In similar situations, many clinicians would have recommended palliative measures. However, the case presented specific factors that justified our approach. First, all the cardiac and noncardiac abnormalities were newly discovered in a patient without any significant previous medical history. Second, our initial goal was to alleviate the tricuspid valve obstruction to allow time for a definitive diagnosis. Third, as mentioned previously, we believed that the large-bore vacuum-assisted aspiration thrombectomy system could facilitate a rapid tissue diagnosis. Finally, the family expressed a strong desire for aggressive intervention and requested all available options to guide the patient’s oncologic management.

This case report further augments the evolving evidence supporting the combined use of large-bore vacuum-assisted aspiration thrombectomy system and ECMO for managing critically unstable patients. In such intricate scenarios, the priority is on hemodynamic stabilization through ECMO, which then enables more definitive interventions such as intracardiac tumor debulking. Moreover, the favorable outcome of this case underscores the benefits of a multidisciplinary approach involving cardiologists, cardiac surgeons, oncologists, and critical care specialists to ensure timely resuscitation, establish a definitive diagnosis, and devise a more comprehensive treatment plan.

## Funding Support and Author Disclosures

The authors have reported that they have no relationships relevant to the contents of this paper to disclose.

## Appendix

Video 1 Apical 4-Chamber View on Transthoracic Echocardiography Revealing Before and After Treatment (A) Before treatment. (B) After treatment with aspiration thrombectomy and rituximab, cyclophosphamide, doxorubicin (hydroxydaunomycin), vincristine (Oncovin), and prednisone (R-CHOP) therapy.

Video 2 Intraprocedural Transesophageal Echocardiography Revealing the Extent of Tumor Aspirated (A) Tumor characteristics. (B) Tumor removal.
